# Binding and neutralizing antibody responses to SARS-CoV-2 in very young children exceed those in adults

**DOI:** 10.1172/jci.insight.157963

**Published:** 2022-04-22

**Authors:** Ruth A. Karron, Maria Garcia Quesada, Elizabeth A. Schappell, Stephen D. Schmidt, Maria Deloria Knoll, Marissa K. Hetrich, Vic Veguilla, Nicole Doria-Rose, Fatimah S. Dawood, SEARCh Study Team

**Affiliations:** 1 Department of International Health, Johns Hopkins Bloomberg School of Public Health, Baltimore, Maryland, USA.; 2 Vaccine Research Center, National Institute of Allergy and Infectious Diseases, NIH, Bethesda, Maryland, USA.; 3 CDC, Atlanta, Georgia.; 4 The SEARCh Study Team is detailed in Supplemental Acknowledgments.

**Keywords:** COVID-19, Infectious disease, Adaptive immunity

## Abstract

**Background:**

SARS-CoV-2 infections are frequently milder in children than adults, suggesting that immune responses may vary with age. However, information is limited regarding SARS-CoV-2 immune responses in young children.

**Methods:**

We compared receptor binding domain–binding antibody (RBDAb) titers and SARS-CoV-2–neutralizing antibody titers, measured by pseudovirus-neutralizing antibody assay in serum specimens obtained from children aged 0–4 years and 5–17 years and in adults aged 18–62 years at the time of enrollment in a prospective longitudinal household study of SARS-CoV-2 infection.

**Results:**

Among 56 seropositive participants at enrollment, children aged 0–4 years had more than 10-fold higher RBDAb titers than adults (416 vs. 31, *P <* 0.0001) and the highest RBDAb titers in 11 of 12 households with seropositive children and adults. Children aged 0–4 years had only 2-fold higher neutralizing antibody than adults, resulting in higher binding-to-neutralizing antibody ratios compared with adults (2.36 vs. 0.35 for ID_50_, *P =* 0.0004).

**Conclusion:**

These findings suggest that young children mount robust antibody responses to SARS-CoV-2 following community infections. Additionally, these results support using neutralizing antibody to measure the immunogenicity of COVID-19 vaccines in children aged 0–4 years.

**Funding:**

CDC (award 75D30120C08737).

## Introduction

Our understanding of the epidemiology of SARS CoV-2 infection in children has evolved since late 2019. Early in the pandemic, SARS-CoV-2 infections were diagnosed less frequently in children than in adults; this raised questions about whether children were less susceptible to infection. Studies have since documented that children can be infected at similar rates as adults (1) and can transmit infection (2–4). Although children can develop severe COVID-19, they are more likely than adults to be asymptomatic or mildly symptomatic (5, 6), suggesting that the immunologic response to infection may vary with age. Data are mixed as to whether children mount more robust SARS-CoV-2 antibody responses than adults following infection (7–9). Information about children aged 0–4 years is especially limited, with relatively small case series reported. While COVID-19 vaccines are recommended for children aged 5–17 years, evaluation of several vaccines in children aged 0–4 years is ongoing. Assessment of the magnitude and quality of the antibody responses to SARS-CoV-2 infection in very young children could inform COVID-19 vaccine assessment and deployment in this age group. Determination of the magnitude of SARS-CoV-2 receptor binding domain (RBD) antibody relative to neutralizing antibody may be useful, as a predominance of binding relative to neutralizing antibody has been observed in response to some other viral infections and vaccines, which, in some instances, may diminish protective immunity (10–12). Moreover, differences in binding-to-neutralizing antibody (B/N) ratios have been observed when comparing responses to SARS-CoV-2 infection and vaccination (13).

The SARS-CoV-2 Epidemiology And Response in Children (SEARCh) study is a prospective household cohort study designed to address SARS-CoV-2 susceptibility, illness, transmission, and immunologic responses in children aged 0–4 years and their household members (see Methods). In this cross-sectional analysis of enrollment sera, we compare titers of SARS-CoV-2 binding and neutralizing antibody to WT SARS-CoV-2 and the Delta variant in adults, children aged 5–17 years, and children aged 0–4 years.

## Results

**Figure 1:**
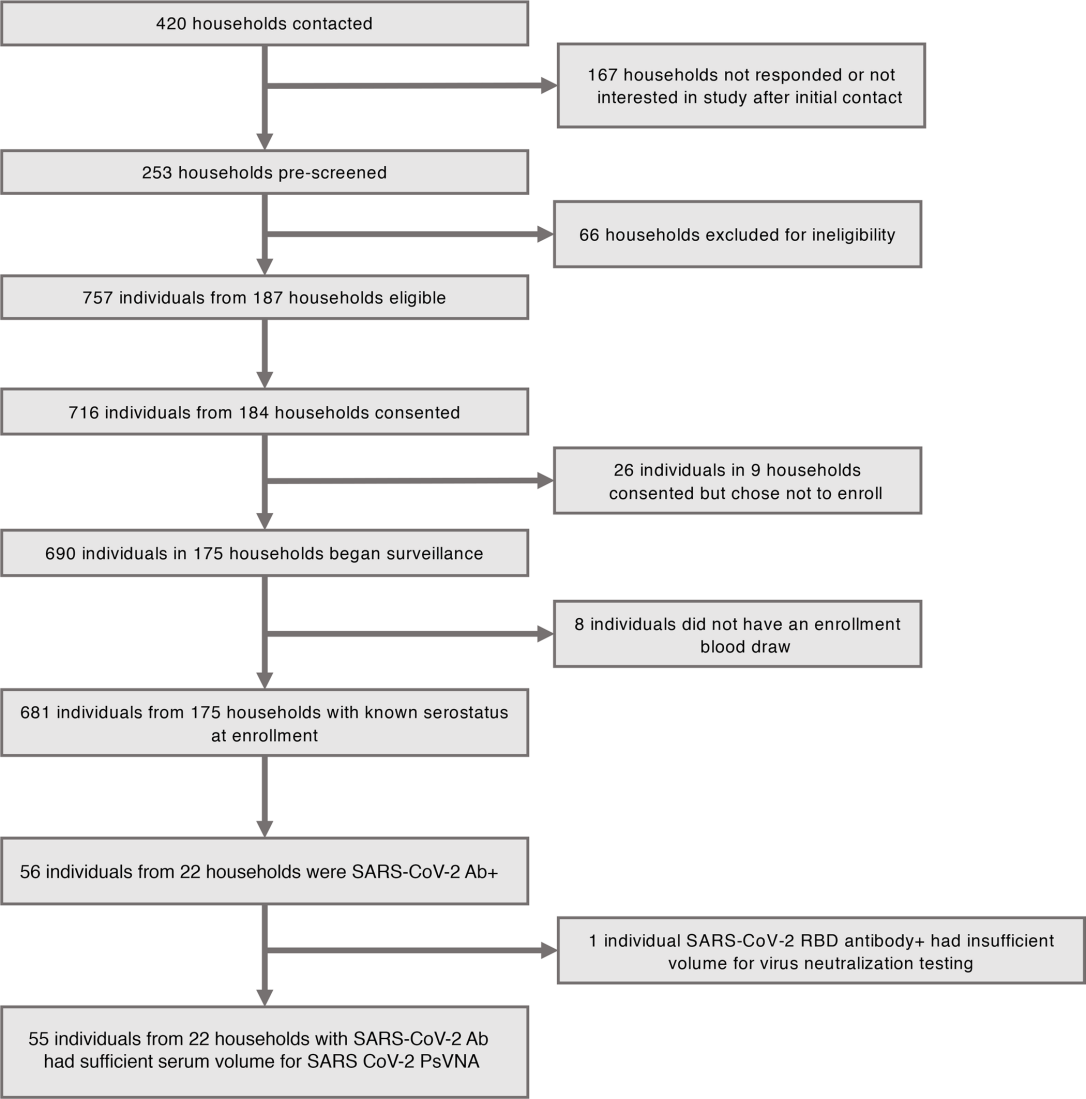
Households contacted, screened, and enrolled in SEARCh, and numbers of participants who were SARS-CoV-2 seropositive at the time of enrollment.

Sera were collected from 682 SEARCh study participants in 175 households, including 332 (49%) adults aged 18–62 years, 96 (14%) children aged 5–17 years, and 254 (37%) children aged 0–4 years (Figure 1). We detected RBD antibody (RBDAb) in sera from 56 (8%) participants in 22 households, including 28 RBDAb-seropositive children (Figure 1); demographic characteristics of these SARS-CoV-2–seropositive participants are shown in Table 1. The proportion of RBDAb-seropositive participants that reported suspected COVID-19 prior to enrollment did not vary significantly by age: 8 of 15 (53%) children aged 0–4 years, 5 of 13 (38%) children aged 5–17 years, and 20 of 28 (75%) adults (*P =* 0.2006 for adults vs. all children). In 8 households with more than 1 member with prior suspected SARS-CoV-2 infection, all suspected infections were reported to have occurred within 1 calendar week of each other. None of the participants was hospitalized with COVID-19 disease before enrollment.

**Figure 2:**
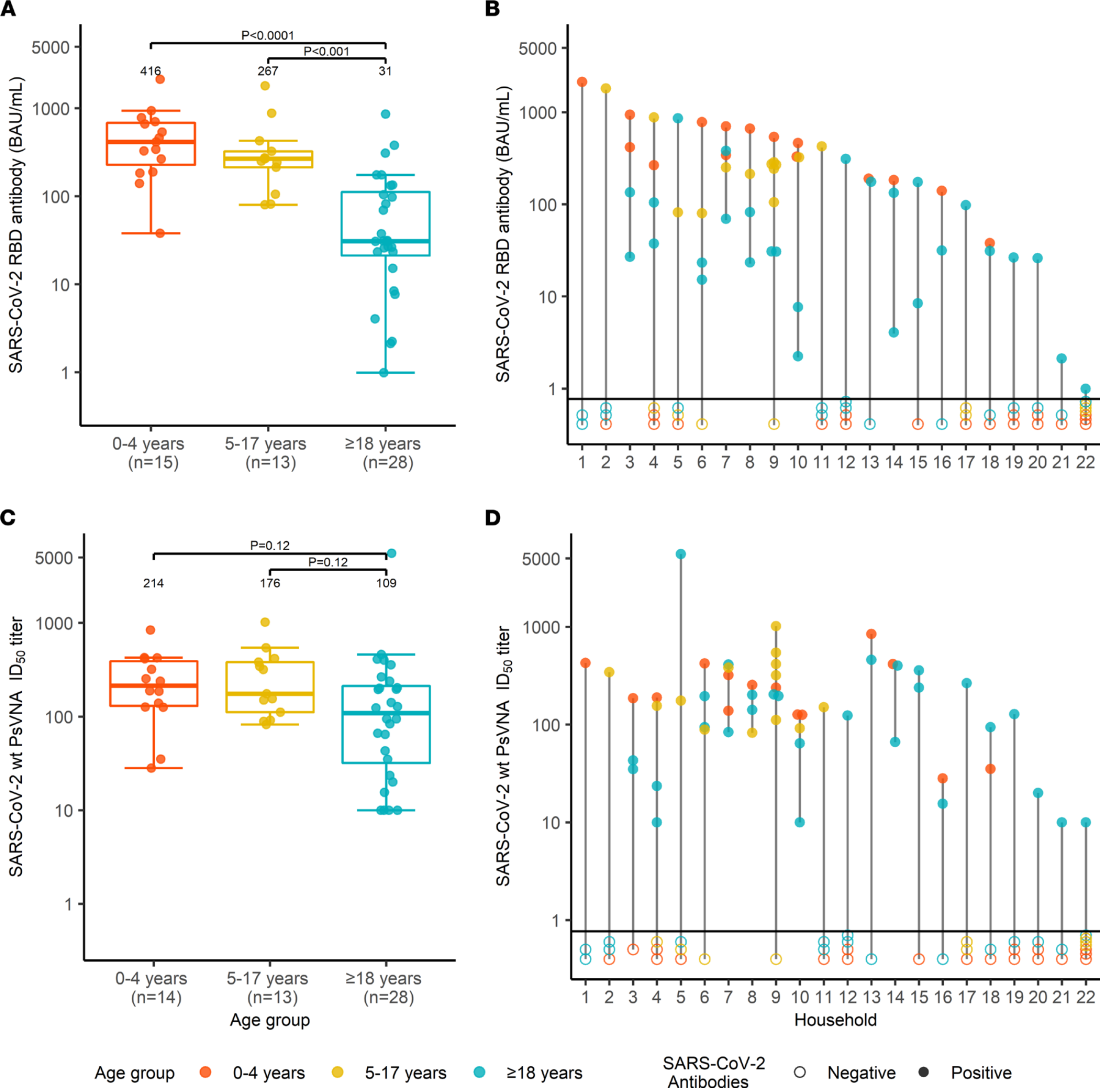
SARS-CoV-2 serum antibody titers at enrollment in SEARCh. (**A**) Box-and-whiskers plot of SARS-CoV-2 RBDAb titers (BAU/mL) by age (orange, children 0–4 years; yellow, children 5–17 years; blue, adults). Bars indicate median values, box bounds represent IQRs, whiskers illustrate variability in relation to the IQR, and outliers are depicted beyond the whiskers. (**B**) RBDAb titers by household. Color scheme is the same as for **A**; filled circles represent RBDAb titers in seropositive individuals, and open circles represent RBD-seronegative household members not included in the analysis. The horizontal line distinguishes between the 2 groups. (**C**) WT SARS-CoV-2–neutralizing antibody ID_50_ titers measured by pseudotyped lentivirus reporter neutralization assay (PsVNA), shown and summarized by age group, as in Figure 1A. Samples with titers below the level of detection were assigned a value of 10. (**D**) PsVNA ID_50_ titers by household, using the sequence and color scheme shown for **B**. Statistical differences in antibody titers were compared using the 2-sided Mann-Whitney-Wilcoxon test. To adjust for 3 age group comparisons for each analysis in **A** and **C**, Bonferroni’s correction was applied, and the *P* value was multiplied by 2. One specimen from a child in the 0–4 year age group was not tested for neutralizing antibodies owing to insufficient sample volume.

Among the 56 participants who were RBDAb seropositive, the median titer of RBDAb (binding antibody units [BAU]/mL) was more than 10-fold higher in children aged 0–4 years than in adults (416 [IQR = 228–683] vs. 31 [IQR = 21–112]), *P <* 0.0001) (Figure 2A); children aged 5–17 years also had higher RBDAb titers than adults (267 [IQR = 213–324] vs. 31 [IQR = 21–112] *P =* 0.0001, Figure 2A). When adults from 7 households without SARS-CoV-2-seropositive children were excluded, the median RBDAb titer for adults was again 31 (IQR = 23–112), and differences between RBDAb titers in adults and in children aged 0–4 years and 5–17 years remained significant (*P <* 0.0001 and *P* = 0.0004, respectively; data not shown).Thirteen of 22 (59.1%) households had more than 1 member who was seropositive for RBDAb; all enrolled household members were seropositive in 5 of 22 households (22.7%) (Figure 2B). Children aged 0–4 years had the highest RBDAb titers in 11 of 12 households with a seropositive child aged 0–4 years (Figure 2B).

**Figure 3:**
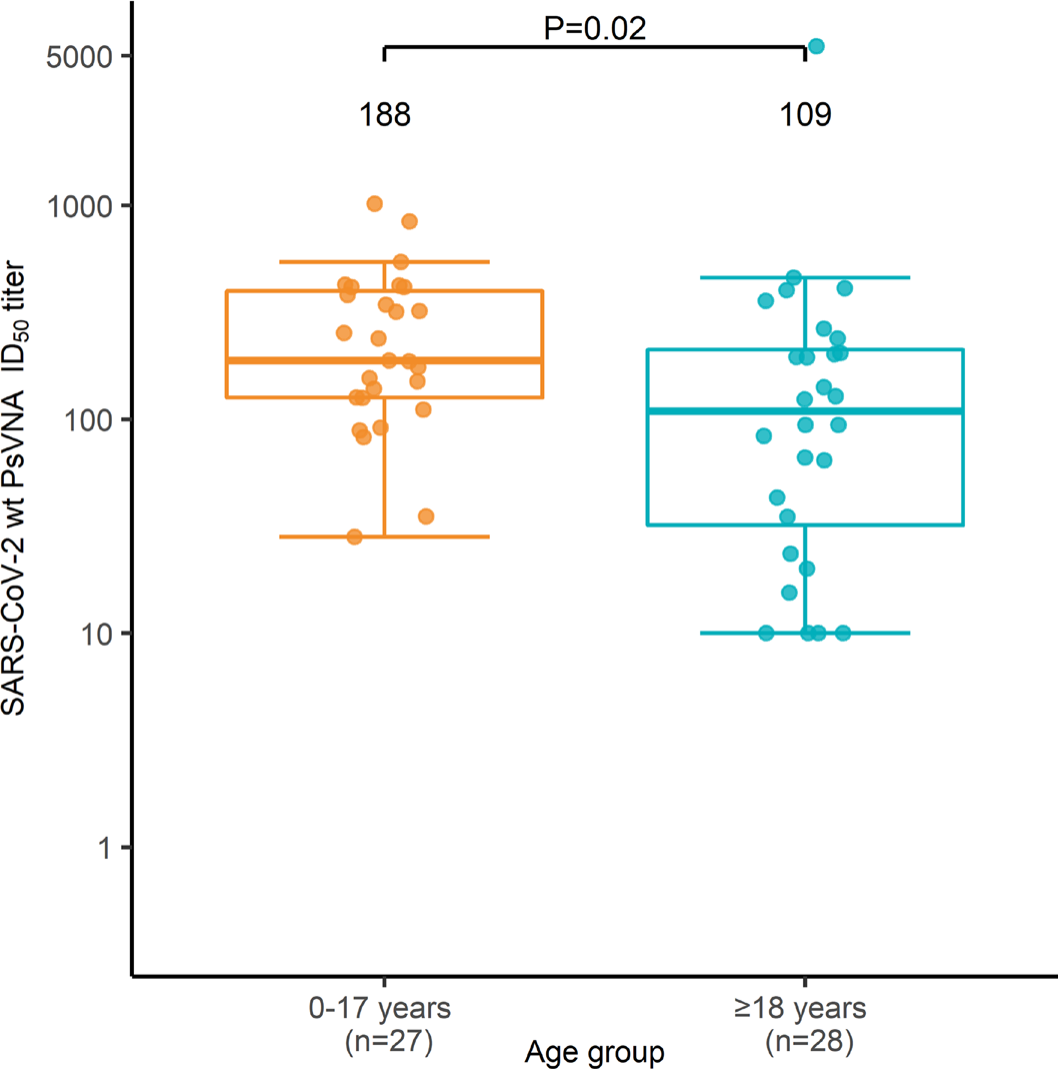
Box-and-whiskers plot of SARS-CoV-2 WT PsVNA ID_50_ titers for all children ages 0–17 versus adults. Statistical differences in antibody titers for children ages 0–17 (light orange) and adults (blue) were compared using the 2-sided Mann-Whitney-Wilcoxon test. Bars indicate median values, box bounds represent the IQRs, whiskers illustrate variability in relation to the IQR, and outliers are depicted beyond the whiskers. PsVNA, pseudotyped lentivirus reporter neutralization assay.

Children aged 0–4 and 5–17 years had similar pseudotyped lentivirus reporter neutralization assay (PsVNA) 50% inhibitory dilution (ID_50_) titers against WT SARS-CoV-2 (Figure 2C). Overall, PsVNA ID_50_ titers among children aged 0–17 years were nearly 2-fold higher than among adults (188 [IQR = 126–398] vs. 109 [IQR = 32–212], *P =* 0.02) (Figure 3). Children aged 0–4 years also had the highest PsVNA ID_50_ titers of all seropositive household members in 9 of 12 households (Figure 2D). PsVNA ID_80_ titers showed similar patterns by age and household (Supplemental Figure 1; supplemental material available online with this article; https://doi.org/10.1172/jci.insight.157963DS1). PsVNA ID_50_ neutralizing antibody titers against the Delta variant of SARS-CoV-2 were comparatively modest in all age groups (children aged 0–4, 214 [IQR = 130–391] WT vs. 81 [IQR = 23–116] Delta; children aged 5–17, 176 [IQR = 111–382] WT vs. 64 [IQR = 23–226] Delta; adults, 109 [IQR = 32–212] WT vs. 24 [IQR = 10–84] Delta; Supplemental Figure 2) and the ratio of titers against WT SARS-CoV-2 and the Delta variant did not differ between age groups.

**Figure 4:**
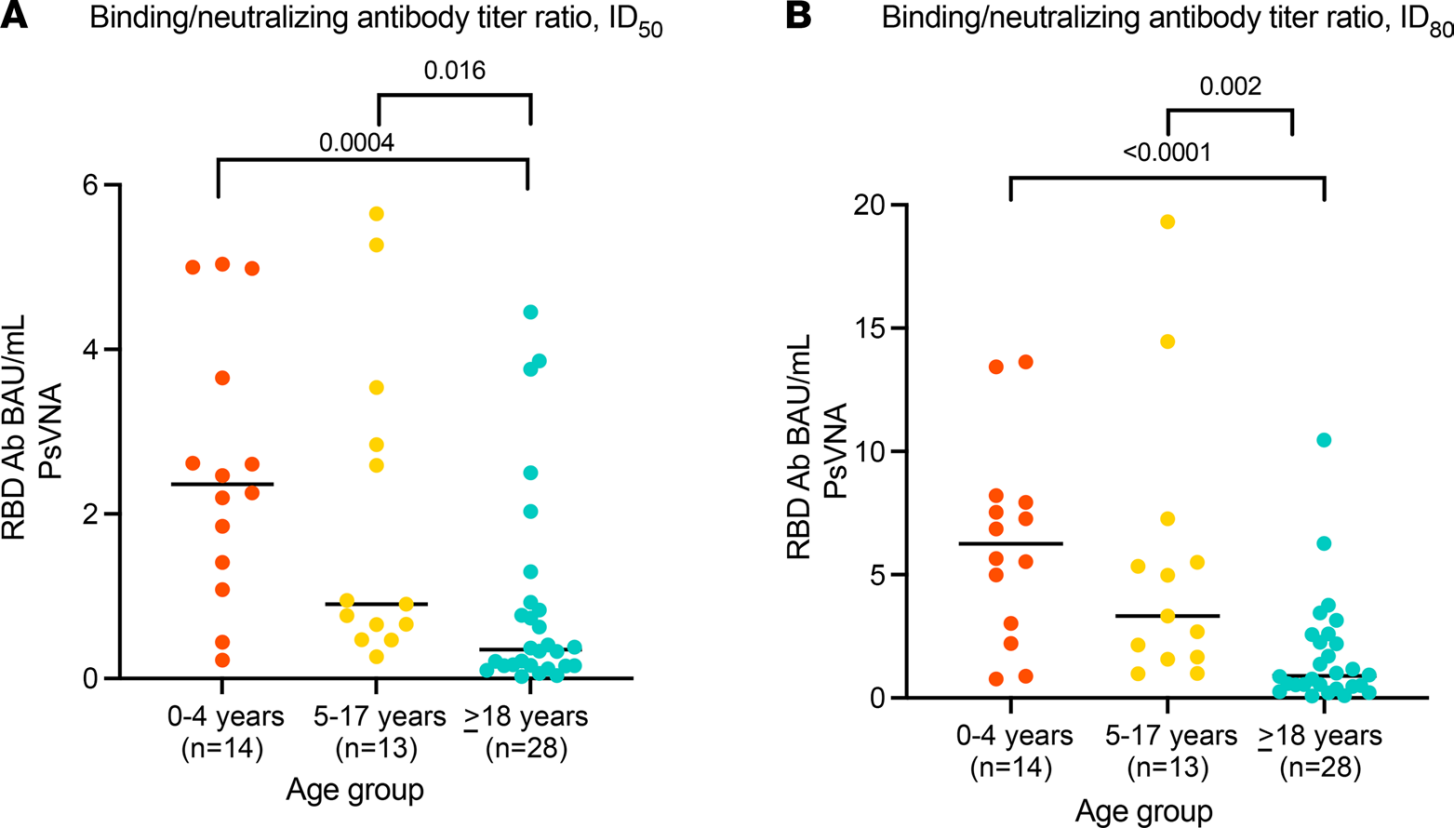
Ratios of SARS-CoV-2 RBDAb to SARS-CoV-2 PsVNA. (**A**) ID_50_ titers. (**B**) ID_80_ titers. Statistical differences in ratios of SARS-CoV-2 RBDAb to SARS-CoV-2 PsVNA (orange, children 0–4 years; yellow, children 5–17 years; blue, adults 18–62 years) were compared using the 2-sided Mann-Whitney-Wilcoxon test. To adjust for 3 age group comparisons for each analysis, Bonferroni’s correction was applied, and the *P* value was multiplied by 2. Bars indicate median values. RBDAb, receptor binding domain antibody; PsVNA, pseudotyped lentivirus reporter neutralization assay.

The median (IQR) B/N ratio using PsVNA ID_50_ was highest in children aged 0–4 years (2.36 [IQR = 1.33–3.99]), followed by children aged 5–17 years (0.9 [IQR = 0.56–3.19]), and lowest in adults (0.35 [IQR = 0.15–0.90]) (*P =* 0.0004 for children aged 0–4 years vs. adults; *P =* 0.016 for children 5–17 years vs. adults) (Figure 4A). Trends were similar for the B/N ratio using PsVNA ID_80_ (Figure 4B).

## Discussion

In this analysis of 56 adults and children with serologic evidence of prior SARS-CoV-2 infection, children aged 0–4 years had approximately 10-fold higher levels of RBDAb and approximately 2-fold higher levels of neutralizing antibody against WT SARS-CoV-2 compared with those of adults. The consistency of these findings within households suggests that the differences were likely unrelated to timing of infection, as household members would likely have been infected with SARS-CoV-2 at approximately the same time. Differences in the relative magnitude of antibody response between children and adults were also unlikely to result from differences in severity of illness (9), as many children had no known history of COVID-19, suggesting they experienced mild or subclinical infection.

Relatively few studies have directly compared the antibody responses to SARS-CoV-2 in children and adults, particularly between children aged 0–4 years and adults. A study of hospitalized patients found that adults mounted higher neutralizing antibody responses than children. (7) In contrast, one community-based study of household clusters of mild COVID-19 found that children had higher and more sustained titers of SARS-CoV-2–neutralizing antibody than adults (8). Another community-based study found that children had higher titers of binding IgG antibody against SARS-CoV-2 spike, nucleocapsid, and RBD and similar neutralizing antibody levels compared with adults (9). Our findings expand upon these community-based studies by (a) contributing additional data for children aged 0–4 years, a relatively understudied population with respect to immune responses to SARS-CoV-2; (b) demonstrating that differences in the magnitude of the RBDAb titers are consistent within households in which timing of infections is likely similar; and (c) demonstrating that although children generally develop significantly higher titers of neutralizing antibody than adults, very young children appear to make proportionally more SARS-CoV-2 RBDAb than neutralizing antibody, as evidenced by a higher B/N geometric mean titer ratio in this age group compared with adults. It is possible that these robust humoral immune responses diminish rates of serious or highly symptomatic infection by promoting viral clearance. Our findings are also consistent with the observation that children aged 5–11 years and adults develop comparable SARS-CoV-2 antibody responses to the BNT162b2 vaccine when children aged 5–11 years receive one-third of the dose given to adults (14).

We observed a stepwise downward progression in B/N geometric mean titer ratio with age: children aged 0–4 years had the highest ratio of B/N antibody, children aged 5–17 years had an intermediate level, and adults had the lowest. The finding that young children had relatively high titers of binding antibody relative to neutralizing antibody was unexpected; the reasons for it are unknown. One possibility is that children aged 0–4 years have had fewer opportunities for priming infections with related betacoronaviruses (15) and, therefore, may take longer to develop high-affinity neutralizing antibody than coronavirus-experienced older children and adults. While cross-reactive antibodies against SARS-CoV-2 have been described in older children and adults, they have been less frequently detected in very young children (16, 17). The pattern of neutralizing antibody epitope recognition may also differ in young children and adults, as observed for other respiratory viruses (11). For example, adults may be more likely to recognize neutralizing epitopes in S2 or N-terminal domain than young children. Children may also have more durable RBD-specific antibody responses (9). The kinetics of the SARS-CoV-2 RBD and neutralizing antibody responses in each age group will be investigated using longitudinal samples obtained in the SEARCh study.

This study has several limitations. First, the number of SARS-CoV-2 RBDAb-positive individuals was relatively small, which may limit interpretation of the results. Second, findings in this analysis likely largely reflect humoral immune responses to WT-like SARS-CoV-2 infections and may not be generalizable to infections with emerging variants. Third, this was a cross-sectional analysis, and it may not have fully accounted for potential differences in timing of infection between adults and children. However, data from households in which the timing of suspected COVID-19 infection was reported indicate that these events occurred in close temporal proximity. Additional longitudinal studies are needed to confirm our initial observations in larger groups of individuals in whom timing of infection is known and who were infected with other SARS-CoV-2 variants.

We have shown that young children aged 0–4 years who were infected with SARS-CoV-2 developed substantial RBD binding and neutralizing antibody responses to WT SARS-CoV-2, and that their immune responses frequently exceeded those of adults in the same households. The differences in B/N ratios by age suggest that RBDAb responses may not predict neutralizing antibody responses as reliably in young children as in adults (18) and that neutralizing antibody responses should continue to be assessed in vaccine trials involving this youngest age group.

## Methods

### Participants.

Sera tested in this study were obtained at enrollment from individuals participating in SEARCh study, a longitudinal household-based cohort study in Maryland, designed to examine the epidemiology and immune response to SARS CoV-2 infection. Households were eligible if they contained at least 1 child aged 0–4 years; individuals were eligible if they had not yet received a COVID-19 vaccine. SEARCh study participants were enrolled between November 2020 and March 2021. Participants were followed prospectively for evidence of SARS-CoV-2 infection using molecular and serologic diagnostic techniques for 8 months or through October 2021, whichever occurred sooner. These prospective data will be the subject of a future report. Participants or their parents/guardians were asked whether they were told by a healthcare provider that they had suspected or confirmed COVID-19 prior to enrollment. Household members also completed questionnaires throughout the study that included information on household composition, illness symptoms, and school, work, and leisure activities. Data were collected using Research Electronic Data Capture (REDCap).

### Antibody assays.

Enrollment sera were first tested for antibody against the RBD of the WT (WA1) strain of SARS-CoV-2 using the Roche Elecsys Anti-SARS-CoV-2 S electrochemiluminescence immunoassay (Elecsys Anti-SARS-CoV-2) in the Department of Pathology, Johns Hopkins School of Medicine. The correlation between the WHO standard and the Roche Elecsys Anti-SARS-CoV-2 is 0.9996 (Pearson’s *r*^2^), and the conversion factor between this assay and the WHO standard is 1.0288. RBD antibody titers are expressed as BAU/mL. Sera that were RBD antibody positive were tested for neutralizing antibody using a pseudotyped reporter neutralization assay (PsVNA). Neutralization of WT (WA1.D614G) SARS-CoV-2 and of the Delta variant (B.1.617.2) of SARS-CoV-2 were measured at the Vaccine Research Center of the US NIH in a single-round-of-infection assay with lentivirus-based pseudotyped virus particles (pseudoviruses) as previously described (19, 20). The ID_50_ and ID_80_ titers are reported. ID_50_ is considered a more sensitive measure of neutralization, and it is the customary metric in SARS-CoV-2 publications; ID_80_ is considered a more stringent measure. Median RBD antibody titers and neutralizing antibody titers were compared by age group among all RBD antibody–seropositive participants. Because SARS-CoV-2 infections may have occurred at any point prior to cohort enrollment and antibody titers may wane over time, individual RBD antibody and neutralizing antibody titers were also compared by age group within households, with the assumption that infections within households occurred at around the same time. The ratio of RBD binding antibody to WT neutralizing antibody was calculated for each participant, and the median B/N ratio was calculated for each age group.

### Statistics.

Analyses were performed using Graphpad Prism and R version 4.1.0 (R Foundation for Statistical Computing) in RStudio version 1.4.1717. A *P* value of less than 0.05 defined statistical significance. Bonferroni’s correction was used to adjust *P* values for multiple comparisons between age groups. Antibody titers were summarized as medians because the data were not normally distributed. One specimen from a child in the 0–4 year age group was not tested for neutralizing antibodies owing to insufficient sample volume. Statistical differences in antibody titers were compared using the 2-sided Mann-Whitney-Wilcoxon test, and proportions were compared using 2-sided Fisher’s exact test. Figures were generated using Graphpad Prism and the Ggplot2 package.

### Study approval.

Prior to study participation, all participants or their parents/guardians provided informed consent, and children aged 7 years and older also provided assent. The study protocol was reviewed and approved by the Johns Hopkins Bloomberg School of Public Health Institutional Review Board (IRB00014200).

## Author contributions

RAK, VV, and FSD conceived and designed the study. EAS managed the study and acquired data. SDS and NDR performed and analyzed neutralizing antibody assays. MGQ, MKH, and MDK performed data analysis. All authors contributed intellectually and read, edited, and approved the final manuscript.

## Supplementary Material

Supplemental data

ICMJE disclosure forms

## Figures and Tables

**Table 1:**
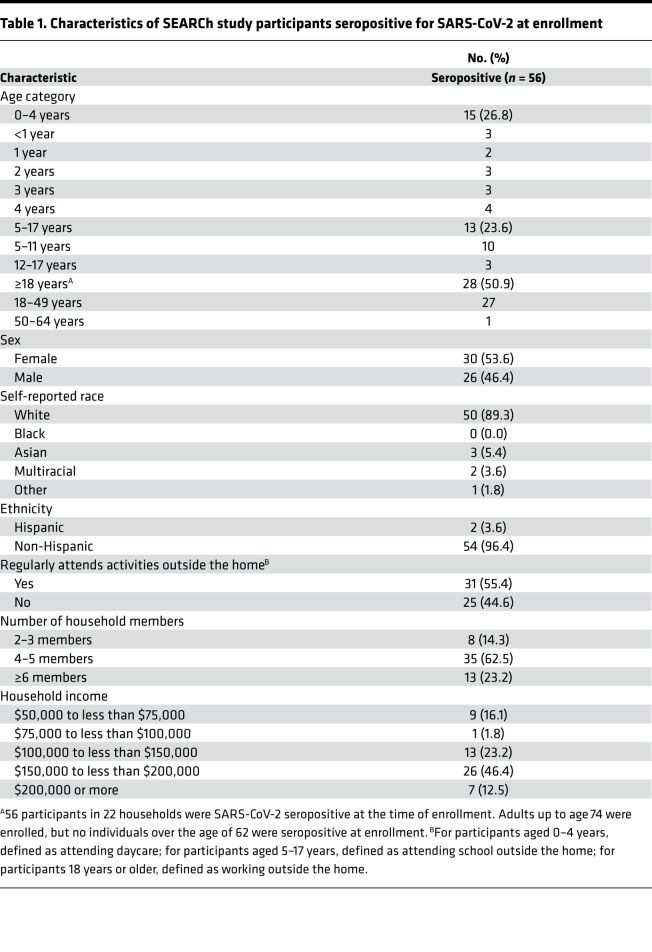
Characteristics of SEARCh study participants seropositive for SARS-CoV-2 at enrollment

## Version 1 03/22/2022

In-Press Preview

## Version 2 04/22/2022

Electronic publication
