# Supplementary material for: Binding and neutralizing antibody responses to SARS-CoV-2 in very young children exceed those in adults
Source: JCI Insight. 2022 Apr 22;7(8):e157963. doi: 10.1172/jci.insight.157963 (PMC9089786; doi:10.1172/jci.insight.157963)
Supplement: Supplemental data [file jciinsight-7-157963-s133.pdf]

Supplemental Figures

Figure S1

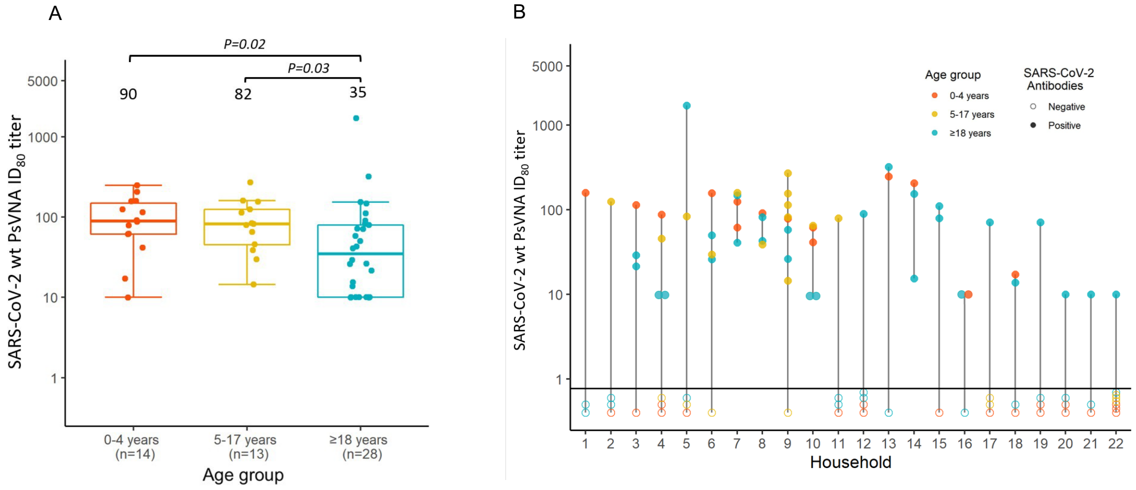

Figure S1A. wt SARS-CoV-2 neutralizing antibody ID_{80} titers measured by pseudovirus viral neutralizing antibody (PsVNA); shown and summarized by age group as in Figure 1A. Samples with titers below the level of detection were assigned a value of 10. **Figure S1B:** PsVNA ID_{80} titers by household, using the sequence and color scheme shown for Figure 1B. Statistical differences in antibody titers were compared using the Mann-Whitney-Wilcoxon test (two-sided). A Bonferroni correction was applied to adjust for 3 age group comparisons and the p-value was multiplied by 2. One specimen from a child in the 0-4 years age group was not tested for neutralizing antibodies due to insufficient sample volume.

Figure S2

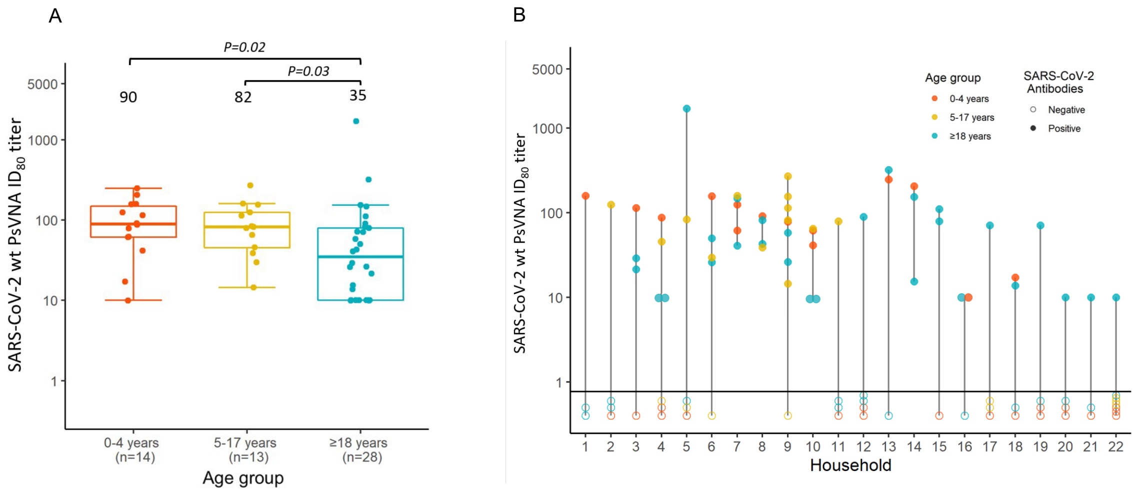

Figure S2. SARS-CoV-2 neutralizing antibody ID_{50} and ID_{80} titers to the Delta variant; measured by pseudovirus viral neutralizing antibody (PsVNA). Data are shown and summarized by age group as in Figure 1A. Samples with titers below the level of detection were assigned a value of 10. **Figure S2A:** PsVNA ID_{50} titers **Figure S2B:** PsVNA ID_{80} titers. Data are shown and summarized by age group as in Figure 1A. Statistical differences in antibody titers were compared using the Mann-Whitney-Wilcoxon test (two-sided). A Bonferroni correction was applied to adjust for 3 age group comparisons and the p-value was multiplied by 2. One specimen from a child in the 0-4 year age group was not tested for neutralizing antibodies due to insufficient sample volume.

SEARCh Study Team*

*Walker Black[3], Christine Council-DiBitetto[1], Tina Ghasri[1], Amanda Gormley[1], Milena Gatto[1], Maria Jordan[1], Karen Loehr[1], Jason Morsell[1], Jennifer Oliva[1], Jocelyn San Mateo[1], Khadija Smith[1], Kimberli Wanionek[1], Cathleen Weadon[1], Suzanne Woods[1]

Affiliations

1. Department of International Health, Johns Hopkins Bloomberg School of Public Health, Baltimore, MD
2. Vaccine Research Center, National Institute of Allergy and Infectious Diseases, National Institutes of Health, Bethesda, MD
3. Centers for Disease Control and Prevention, Atlanta, GA
